# On Fracture of the Skull

**Published:** 1855-03

**Authors:** James Syme

**Affiliations:** Professor of Clinical Surgery in the University of Edinburgh


### RECORD OF MEDICAL SCIENCE.

*On Fracture of the Skull.* By JAMES SYME, Esq., Professor of Clinical Surgery in the University of Edinburgh.

This young man, John W——, aged twenty-three, was at work at a flour-mill in Kirkaldy on the 25th of July last, when a fourteen pound weight fell from the fourth story, and struck him on the head, about an inch and a half from the vertex, where you now observe a depressed cicatrix. On his admission here, I found a depressed fracture of limited extent, and, on removing with bone-pliers the fragments of the outer table, discovered a circular portion of the inner table, of much larger size, which I succeeded in extracting piecemeal through the opening in the outer table. He was conscious at the time, and has ever since been in the full enjoyment of his intellectual faculties. The wound was simply dressed, and went on favorably until its contraction produced inversion of the edges of the scalp, when the hairs growing into the wound prevented further cicatrization, till I pared away the inverted edge a few weeks ago, after which the wound soon healed completely.

In such cases as this there is no reproduction of bone in the central part of the cicatrix, which is intimately adherent to the dura mater; and though it becomes after a time exceedingly dense, yet it is prudent for the patient to wear some sort of protection if liable to be exposed to external violence.

I may take this opportunity, gentlemen, of remarking that injuries of the head have long been a fruitful subject of bad practice and traditional error. In the olden times injury of the head, accompanied with insensibility, was thought to require scalping of the part, so as to allow a thorough examination of the bone, when, if a fissure was discovered, the trephine was applied, under the idea that noxious transudations were liable to pass from without inwards. Even in cases where there was no evidence of cerebral disorder, it was the practice to remove any flap of the scalp that might have been torn up from the cranium. So lately as the time of Charles II., we find it mentioned in the valuable cases recorded by Wiseman, then sergeant-surgeon, that a surgeon who had cut off a large flap of skin in these circumstances was so proud of his performance, that he hung up the piece of scalp in his shop, to show what a great operator he was. Even in recent times, whenever injury of the head produced loss of consciousness, it was the practice to bleed largely immediately after the accident.

All these errors have now been disposed of, but something still remains to be cleared away. You have, no doubt, all of you heard of the operation of trephining, and have read that it is required under the following circumstances: first, when purulent fluid exists between the bone and the dura mater; second, when blood has been effused from the main artery of the dura mater; third, when the bone is depressed injuriously: and you will perhaps be somewhat surprised when I tell you that, if you except the case of punctured fracture of very small size, such as a sharp-pointed instrument would produce, trephining is never of any service. Take first the cases of effusion of matter and of blood. Though I have been now nearly thirty-five years connected, in one way or other, with this hospital, I have never known a single case where blood or matter has been withdrawn with a satisfactory result; indeed, I have never seen blood withdrawn at all by trephining, nor did I ever find a surgeon who had met with such a case. You will therefore do well to regard trephining under these circumstances as an operation that does not at all concern you. Also in depressed fracture, if you except the special case of small puncture, you will find trephining never necessary. If the bone is broken, it is so much so that it can be elevated by bone-pliers. Make a crucial incision in the scalp, and raise the flaps; try different parts with the bone-pliers till you can raise a small portion; cut off bits of the overhanging edge, if necessary, and you will be able to complete the elevation of the whole. If the bone has not injured the dura mater, the patients generally recover; if the longitudinal sinus is injured, they die, so far as my experience goes, although there appears no special reason why injury of this venous channel should be so much more serious than that of others, and cases of recovery have been recorded. If the dura mater has been injured so as to expose the brain, the case is very unfavorable; and if the brain itself is injured, it is almost hopeless. I say, *almost* hopeless, for it is not entirely so. A little more than a year ago a fire occurred in the Cowgate, which, as you know, is an old street of lofty houses. The occupiers of the fourth story threw out their valuables into the street, and, amongst the rest, a child four years old. It appeared to have fallen on its head, and had injured one parietal bone, over which a soft tumor existed. One day passed, and next day it appeared that some epileptic seizures had occurred, which, on the following day, became almost continuous, the child being scarcely out of one fit before another came on. I thought it right to open the tumor, and on my doing so, blood issued, and a wide crack appeared in the parietal bone, which had given exit to a quantity of cerebral substance, the appearance altogether being so frightful that I did not complete the incision, but regarding the caee as hopeless, sent the child to bed with a piece of wet lint on the wound. However, the convulsive attacks ceased from that time forward; the child showed signs of intelligence within two hours; on the third day it was sitting up, with a smile on its countenance; and in a fortnight after the operation it was running about the ward. I learned a short time since that this child was in the most exuberant health. But such cases are unfortunately quite exceptional.

I may remark, as we have happened to speak on this subject, that epileptic attacks sometimes come on months or years after injuries of the head; and you may perhaps be called upon to "do something for the patient," as it is said—that is to say, to trephine the skull. I have myself on several occasions exposed the bone and removed portions in such cases; but, from the results which have followed, I advise you positively against such a course. The case is different, however, if a sinus continue in connexion with a sharp piece of bone pressing on the brain. I once operated on such a case, and some years afterwards had the satisfaction of seeing the patient in perfect health. The case is thus reported in the tenth Report of the Edinburgh Surgical Hospital for 1833, (*Edinburgh Medical and Surgical Journal*, No. 115.)

Wm. K———, aged twenty-two, from Dunfermline, admitted 3rd September. Between six and seven years ago he suffered a very severe wound of the face from the blasting of a rock. He was at first insensible, and considered in a hopeless state, but gradually recovered so as to be able to follow his employment as a weaver. A small opening, however, between the cheek-bone and ear continued to discharge matter, and he occasionally suffered much pain in his head. During the last four months he had had occasional epileptic fits. On examination, it appeared that the malar bone had been fractured, and the whole upper part of the face was occupied by a dense cicatrix. A piece of bone was felt at the bottom of the sinus, bare, and lying obliquely in respect to the surface of the skull. On the supposition that this was a portion of the cranium which had been depressed at the time of the injury, a crucial incision was made through the cicatrix, to expose the aperture of the skull, which was then enlarged by means of cutting-forceps, so as to admit the extraction of the loose piece. It comprehended both tables of the bone, (temporal,) and was about a third the size of a sixpence. The patient expressed much relief after the operation; but the fits still recurred, though seldom, and a profuse discharge issued from the wound. A probe being gently introduced, entered to the depth of two inches perpendicularly, the substance of the brain seeming to have been hollowed out into an abcess. He returned home, and continues in the same state, though, on the whole, improved.—*London Lancet.*

